# Longitudinal EpiTrack assessment of executive functions following vagus nerve stimulation therapy in patients with drug‐resistant epilepsy

**DOI:** 10.1002/epi4.12855

**Published:** 2023-11-27

**Authors:** Niina Lähde, Pabitra Basnyat, Jani Raitanen, Kai Lehtimäki, Eija Rosti‐Otajärvi, Jukka Peltola

**Affiliations:** ^1^ Department of Neurology Tampere University Hospital Tampere Finland; ^2^ Faculty of Medicine and Health Technology Tampere University Tampere Finland; ^3^ Faculty of Social Sciences, Health Sciences Tampere University Tampere Finland; ^4^ UKK Institute for Health Promotion Research Tampere Finland; ^5^ Department of Neurosurgery Tampere University Hospital Tampere Finland; ^6^ Department of Rehabilitation and Psychosocial Support Tampere University Hospital Tampere Finland

**Keywords:** antiseizure medication, cognition, neuromodulation

## Abstract

**Objective:**

To investigate executive functions and attention with repeated EpiTrack evaluations in a group of DR patients with drug‐resistant epilepsy (DRE) receiving vagus nerve stimulation (VNS) during a follow‐up duration of up to 5 years.

**Methods:**

The study involved 33 patients with DRE who were assessed with EpiTrack as a part of the clinical VNS protocol. Evaluations were scheduled prior to VNS implantation and then at 6 months, 12 months, and yearly thereafter. However, the COVID‐19 pandemic disrupted follow‐up. Therefore, changes in EpiTrack total scores over time were analyzed using a linear mixed‐effects (LMEs) model to compensate for the variation in follow‐up duration when predicting EpiTrack total score changes over 5 years.

**Results:**

The median follow‐up time was 29 months. During each month, the EpiTrack total score was predicted to increase by 0.07 units (95% confidence interval [CI]: 0.01–0.12, *P* = 0.02), corresponding to a change from a baseline score of 27.3 (severe impairment) to a score of 28.9 (mild impairment) at 2 years and a score of 31.5 (almost normal) at 5 years. In the group of patients with psychiatric comorbidities, the EpiTrack total score increased by 0.14 units per month (*P* = 0.003), which was 3.5‐fold higher than the increase of patients without psychiatric comorbidities. For the patients taking 1–2 antiseizure medications (ASMs), the EpiTrack total score increased by 0.11 units per month (*P* = 0.005), which was almost quadruple the rate of patients taking 3–4 ASMs.

**Significance:**

Based on EpiTrack total scores, the LME model predicted a four‐point improvement in executive functions among patients with DRE at 5 years after the initiation of VNS, representing a clinically meaningful change. DRE patients with comorbid depression seemed to experience the most cognitive benefits. In addition, better cognitive outcomes were achieved if the patient took less than three ASMs.

**Plain Language Summary:**

Executive functions and attention may improve during vagus nerve stimulation therapy in patients with drug‐resistant epilepsy. Epilepsy patients who have depression or use fewer than three antiseizure medications are likely to benefit cognitively more from the treatment.

**Key Points**

- In this longitudinal follow‐up study, performance in executive functions improved over time during vagus nerve stimulation (VNS) treatment.
- The EpiTrack performance category changed on average from severely impaired to almost normal.
- Patients with comorbid depression exhibited higher odds of improvement in EpiTrack scores.
- The cognitive response during VNS therapy was superior in patients taking fewer than three antiseizure medications at baseline.

## 1 INTRODUCTION

Deficits in cognitive functioning are common comorbidities in drug‐resistant epilepsy (DRE) that may intensely influence patients' quality of life and daily functioning.^1^ Executive functions and attention are especially often impaired in patients with DRE.^2^ Executive functions refer to high‐order cognitive functions, which, for example, allow a person to adapt to nonroutine situations such as novel, conflicting, or complex tasks.^3^ The cognitive impairments experienced in DRE are usually multifactorial in origin. While some factors, such as etiology and type of epilepsy, are static and cause permanent impairments, others, including side effects of antiseizure medication (ASM), seizures, and psychiatric comorbidities, are dynamic and potentially alleviated by treatment choices.^1^, ^4^

Vagus nerve stimulation (VNS) was approved as an adjunctive treatment for DRE in Europe in 1994 and by the FDA in 1997. It is an alternative treatment option for patients with DRE who are not suitable for resective epilepsy surgery or for whom the surgery has not led to adequate seizure control. The efficacy of VNS in reducing seizure severity and frequency has been established in several randomized controlled trials.^5^, ^6^, ^7^ A recent population‐based study from Norway indicated that the effect of VNS increases over time, and the cumulative probability of ≥50% seizure reduction was as high as 60% after 5 years.^8^

Most of the studies on VNS have focused on determining the seizure outcome, and only limited evidence is available considering the effects of VNS on cognition. The existing data suggest that VNS has positive short‐term effects on working memory performance and visual attention.^9^ Adjunctive VNS treatment may also have long‐term benefits in word retention, short‐term memory, attention, verbal communication, and progress in schoolwork at 1–2 years post‐implantation,^10^, ^11^ but thus far, no convincing evidence of cognitive improvement after long‐term VNS has been reported. In addition, many of the studies on VNS and cognition have focused on memory instead of other cognitive functions, such as executive functions.^12^ The possible positive effects of VNS on cognition and executive functions are likely partially direct and partially indirect, mediated by the changes in seizure frequency and severity, changes in concomitant ASM use, and improvement in mood. The FDA approved VNS for treatment‐resistant depression in 2005 (CE, 2001), and current evidence also supports the positive effect of VNS on mood in patients with DRE.^12^, ^13^, ^14^

EpiTrack is a brief screening tool developed to monitor the cognitive side effects of ASMs on executive functions and attention in patients with epilepsy. This screening tool takes 15 min to complete and consists of six subtests: Trail‐Making Test Parts A and B (TMT‐A and TMT‐B), the interference test, the digit span task (backward), assessment of written word fluency and the maze test.^15^ In our previous study, we utilized EpiTrack to assess attention and executive functions in patients with DRE in consideration for treatment interventions (ASM changes and/or neuromodulation therapies), identifying several clinical features such as the number of ASMs, seizure count, and psychiatric comorbidities, which can have an impact on executive functions in patients with DRE.^16^ Furthermore, we discovered that more than 75% of the patients treated with VNS exhibited deficits in that specific domain prompting us to further investigate whether changes in executive functions occur during VNS treatment in this new study. Unlike extensive neuropsychological examination, EpiTrack is easily and quickly executed, and therefore, suitable for repeated assessments to monitor treatment effects.

The purpose of this study was to investigate executive functions and attention with repeated EpiTrack evaluations in a group of patients with DRE receiving VNS therapy during a follow‐up duration of up to 5 years. In addition, we explored the effect of dynamic factors identified in our previous study on executive functions after initiation of VNS therapy.

## 2 MATERIALS AND METHODS

This was a noninterventional study in which data were collected prospectively but analyzed retrospectively from a VNS quality register at Tampere University Hospital; thus, ethics committee approval was not required according to the Finnish Law on Research. Access to the VNS quality register was granted by the Tampere University Hospital Research, Development and Innovation Centre.

### 2.1 Patients

The patient group consisted of 33 patients with DRE who were implanted with VNS (Model 106 [Aspire®] or Model 1000 [SenTiva®], both from LivaNova PLC, London, UK) at Tampere University Hospital between September 2015 and January 2021. The median duration of VNS was 29 months and ranged from 12 to 60 months. All the patients had previously undergone a presurgical evaluation and were either unsuitable candidates for resective epilepsy surgery or had undergone epilepsy surgery but did not achieve adequate seizure control. Patients with intellectual disabilities were excluded because they were unable to complete the EpiTrack test.

### 2.2 Procedure

We retrospectively extracted information on the etiology and type of epilepsy, age at epilepsy onset, duration of epilepsy, current ASM use, years of education, duration of VNS, psychiatric comorbidities (either at present or in the past), Beck Depression Inventory score at baseline, previous resective epilepsy surgery or other brain surgery, the frequency of predominant seizure type during the 12 months prior to VNS implantation, and 3 months prior to each post‐implantation EpiTrack assessment from the VNS quality register. The predominant seizure type for each patient was defined as the most disabling seizure type noted in the medical records as determined by the physician but not necessarily the most frequent seizure type.^10^ The classification of seizure type was based on video‐electroencephalogram (EEG) findings and seizure semiology. The epilepsy type was classified as temporal lobe epilepsy (TLE), frontal lobe epilepsy (FLE), or other (three multilobar including two frontotemporal and one parieto‐frontal, two multifocal loci, one parietal locus, and one idiopathic generalized epilepsy). The etiology of epilepsy was evaluated based on MRI findings and clinical history. All patients were treated with ASMs in addition to VNS. The number of concomitant ASMs at baseline ranged from 1 to 4. The clinical characteristics of the patients are presented in Table 1.

**TABLE 1:** Demographics and clinical characteristics of the patients.

Total patients (*n* = 33)	Descriptives
Age at baseline in years (median, [IQR])	32 (27–41)
Age at disease onset in years (median, [IQR])	15 (9.5–20)
Sex (female/male)	19/14
Epilepsy duration in years (median, [IQR])	17 (10–24.5)
Epilepsy types (*n*, %)	
Frontal lobe epilepsy	14 (42.4)
Temporal lobe epilepsy	12 (36.4)
Other (including 3 multilobar, 1 parietal, 2 multifocal, and 1 IGE)	7 (21.2)
ILAE Etiology (*n*, %)	
Structural	10 (30.3)
Cortical dysplasia	2 (6.1)
Vascular lesion	3 (9.1)
Cavernoma	1 (3.0)
Av‐malformation	1 (3.0)
Brain trauma	1 (3.0)
Late effects of radiation	1 (3.0)
Hippocampal sclerosis	1 (3.0)
Immune	4 (12.1)
Autoimmune encephalitis	4 (12.1)
Genetic	1 (3.0)
Unknown	18 (54.5)
Previous brain surgery (resective or other)	
Yes (*n*, %)	7 (21.2)
No (*n*, %)	26 (78.8)
Psychiatric comorbidity	
Yes (*n*, %)	9 (27.3)
Present/past	5/4
No (*n*, %)	24 (72.7)
Predominant seizure types at baseline	
FAS	4 (12.1)
FIAS	21 (63.6)
FBTCS	7 (21.2)
Seizure free	1 (3.1)
BDI I at baseline (median, [range])	5 (0–41)
Educational years (median, [range])	12 (9–17)
VNS model (*n*, %)	
1000 (Sentiva®)	10 (30.3)
106 (Aspire®)	23 (69.7)
Number of ASMs at baseline (*n*, %)	
1	1 (3.05)
2	14 (42.4)
3	17 (51.5)
4	1 (3.05)

*Note*: Presented as the median and interquartile range (IQR) (age at baseline, age at onset, and duration of epilepsy) or frequency and proportion (for the rest of the variables).

Abbreviations: ASM, antiseizure medication; BDI, Beck's Depression Inventory; FAS, focal aware seizure; FBTCS, focal to bilateral tonic–clonic seizure; FIAS, focal impaired awareness seizure; VNS, vagal nerve stimulation.

As a part of our clinical VNS protocol, EpiTrack evaluation, and comprehensive neuropsychological and psychiatric assessments were performed for all patients before VNS implantation. Post‐implantation EpiTrack evaluations were conducted at 6 months, 12 months, and thereafter yearly after implantation. Due to the COVID‐19 pandemic, scheduled appointments did not always take place according to our protocol. During follow‐up, nine patients underwent four post‐implantation tests, 16 patients were tested three times, and eight patients were assessed two times. The timing of the assessments is presented in the Figure S1. EpiTrack evaluations were carried out by three epilepsy nurses who were trained by a neuropsychologist (ER‐O). The six subtests included in the EpiTrack evaluation yield an age‐corrected total score with a maximum score of 49 and a minimum score of nine. The cut‐off score for normal performance is 32 points, with scores of 29–31 representing mildly impaired performance, and scores ≤28 representing severely impaired performance. A clinically meaningful change is defined as a four‐point change between two consecutive tests.^15^, ^17^

### 2.3 Statistical analysis

Changes in EpiTrack total scores over time (months) were analyzed using a linear mixed‐effects (LMEs) model with robust standard errors in Stata version 17.0 (StataCorp, College Station, Texas, USA). The equation of the fitted model was

$$\begin{array}{c} Y_{\mathrm{ij}}=\beta_{0}+\beta_{1}\times {\text{time}}_{\mathrm{ij}}+\beta_{2}\times x_{\mathrm{ij}}+\beta_{2}\times x_{\mathrm{ij}}\times {\text{time}}_{\mathrm{ij}}\\ \;+b_{i0}+b_{i1}\times {\text{time}}_{\mathrm{ij}}+\varepsilon_{\mathrm{ij}}, \end{array}$$

where $Y_{\mathrm{ij}}$ is the EpiTrack total score for patient *i* at the *j*th time point, the *β*s are the fixed‐effect coefficients for time and certain covariates (psychiatric comorbidities, number of ASMs, predominant seizure type, and epilepsy type), the *b* terms are the individual random‐effect coefficients assumed to be distributed as ${\left(b_{i0},b_{i1}\right)}_{\sim }^{i.i.d}N\left(0,\sum_{b}\right)$ and $\varepsilon_{\mathrm{ij}}∼N\left(0,\sigma_{\varepsilon }^{2}\right)$ is the error of the *j*th observation for patient *i*. Fixed‐effect coefficients determine the mean EpiTrack total score, and the random effects describe interindividual variation around the mean. Estimates with their 95% confidence intervals (CIs) were calculated. Although the EpiTrack total score is, by definition, already adjusted for age, some of the analyses were adjusted for sex and age whenever appropriate.^15^ Visual representations of the results include observed values of the EpiTrack total score at each time point and fitted average trajectories based on LME models. Moreover, the results of the changes in the EpiTrack total scores over a follow‐up period of up to 5 years are represented by the estimates (with 95% CIs) predicted by the model. *P* values were considered significant at ≤0.05.

## 3 RESULTS

### 3.1 Predicted EpiTrack performance during follow‐up after VNS implantation

**FIGURE 1:**
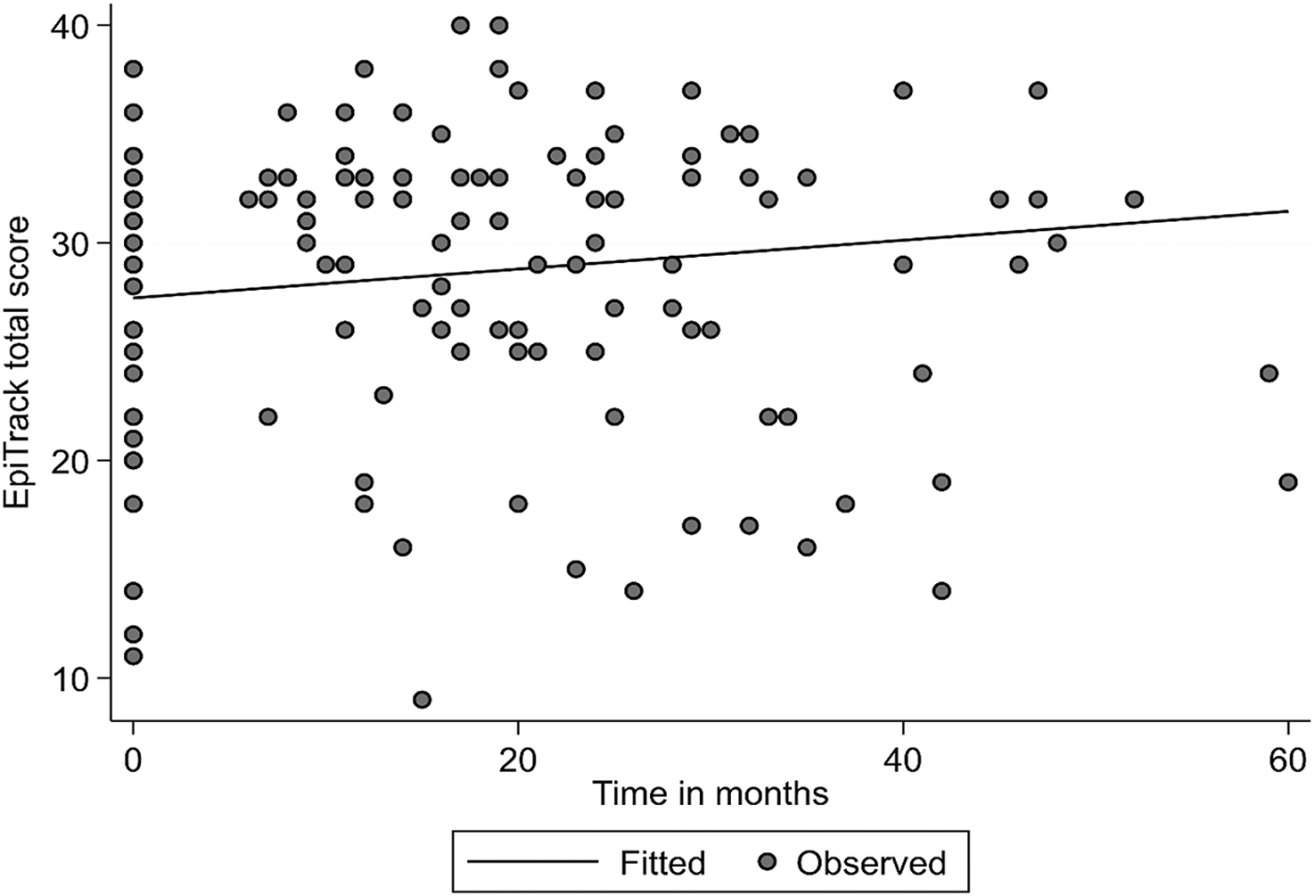
Observed EpiTrack total scores and fitted curve based on linear mixed‐effects model for all patients over time following VNS therapy. VNS, vagus nerve stimulation.

Based on the LME model, there was a significant increase in the EpiTrack total score following VNS therapy during a follow‐up period of up to 60 months. During each month, the EpiTrack total score increased by an average of 0.07 units (95% CI: 0.01–0.12, *P* = 0.02, Figure 1). This corresponds to a change from a baseline score of 27.3 (severe impairment) to a score of 28.9 (mild impairment) at 2 years and a score of 31.5 (almost normal) at 5 years (Table 2).^15^, ^17^ Essential changes to the results were not found after adjusting for sex and age.

**TABLE 2:** Baseline EpiTrack total scores and change (pace of improvement) for all clinical characteristics at 2‐ and 5‐year after the VNS implantation.

Characteristics	Baseline EpiTrack score	Average increase at 2 years^a^	Average increase at 5 years^a^
All patients	27.3	1.6	4.2
Psychiatric comorbidities			
Yes	23	3.3	8.4
No	28.9	0.9	2.4
Predominant seizure types			
FAS/FIAS	27.9	1.4	3.6
FBTCS	25.3	2.8	7.2
ASM			
1–2 ASMs	29.6	2.6	6.6
3–4 ASMs	25.3	0.7	1.8
Epilepsy types			
FLE	26.9	2.6	6.6
TLE	26.8	1.4	3.6
Others	29.8	−0.5	−1.2

Abbreviations: ASM, antiseizure medication; FAS, focal aware seizure; FIAS, focal impaired awareness seizure; FBTCS, focal to bilateral tonic clonic seizure; FLE, frontal lobe epilepsy; TLE, temporal lobe epilepsy.

^a^ Average increase in EpiTrack scores at 2‐ and 5‐year were calculated as average change in EpiTrack score per month obtained from the LME model divided by 24 and 60 months, respectively.

Since the LME model did not allow for analysis of changes in ASMs or seizure frequencies during the follow‐up period, we performed an additional descriptive analysis on individual changes (Tables 3, 4, 5; Figures S2–S5). Remarkably, the EpiTrack performance category at baseline had a major effect on the probability of clinically significant improvement at individual level. Only 1/12 (8.3%) patients with normal performance at baseline experienced clinically significant improvement during follow‐up, whereas 2/8 (25%) patients with mildly impaired performance, and 9/13 (52.4%) patients with severely impaired performance improved significantly during follow‐up. Furthermore, the duration of VNS therapy was at least 2 years for 83.3% of the patients who experienced clinically significant improvement.

**TABLE 3:** Individual changes on ASMs, frequency of predominant seizure type, and EpiTrack total scores during follow‐up in patients with normal EpiTrack performance at baseline.

Sex/age at baseline	Epilepsy type	Psychiatric comorbidity	ASMs at baseline	ASMs at LOCF	Predominant seizure type	Monthly seizure frequency 12 months prior to baseline	Change in predominant seizure type from baseline	Duration of VNS therapy (months)	EpiTrack change
M/53	Other	No	LCM 400, LEV 2000	LCM 400, LEV 2000	FIAS	10	↓ < 25%	13	38 → 40
F/44	TLE	No	BRV 200, LCM 400	BRV 200, LCM 400	FAS	54	↓ < 25%	24	36 → 37
F/33	FLE	No	LTG 500, TPM 400	LTG 500, TPM 50↓	FIAS	0.3	↓ 100%	14	34 → 36
M/22	TLE	No	BRV 200, ESL 1600	BRV 200, ESL 1600	FIAS	12	↓ 100%	26	33 → 35
M/32	FLE	No	LEV 2000, ESL 2000	BRV 200, ESL 2000	FIAS	3	↓ ≥ 75% < 90%	29	32 → 37
F/32	FLE	No	LEV 3000, LCM 600	LEV 3000, LCM 600, CLB 5 +	FBTCS	0.3	No change	29	32 → 33
F/26	Other	No	LEV 3000, LCM 500	LEV 3000, LCM 500	FIAS	0.5	No change	32	32 → 33
F/31	TLE	Yes/past	LCM 400, CLB 50	LCM 400, CLB 35↓, BRV 150 +	FIAS	8	↓ ≥ 25 < 50%	48	32 → 32
F/24	TLE	No	LTG 500, LEV 1750, ZNS 200	LTG 500, LEV 1000↓, ZNS%	FIAS	1	↑ 100%	33	33 → 32
M/29	FLE	No	OXC 2100, ZNS 500, CLB 20	OXC 2100, ZNS 50↓, CLB 20	SF	SF	No change	29	33 → 33
M/29	FLE	No	LCM 600, PER 8, CLB 20	LCM 600, PER 8, CLB 20	FBTCS	0.3	↓ 100%	12	32 → 33
F/22	Other	No	ESL 1600, CLB 15, BRV 200	ESL 1600, CLB 15, BRV 200	FIAS	6	↓ ≥ 25 < 50%	12	34 → 33

*Note*: ↓ = decrease/reduction, ↑ = increase, + = add on, % = withdrawn. The blue boxes in the ‘ASMs at LOCF’ column highlights ASM decrease/reduction, while the red boxes indicate ASM increase. In the ‘Change in predominant seizure type from baseline’ column, blue highlights more than 50% seizure reduction, and red indicates more than 50% seizure increase. In the ‘EpiTrack change’ column, blue highlights patients with clinically significant improvement.

Abbreviations: ASM, antiseizure medication; BRE, brivaracetam; CLB, clobazam; ESL, eslicarbazepine acetate; FAS, focal aware seizure; FBTCS, focal to bilateral tonic clonic seizure; FIAS, focal impaired awareness seizure; FLE, frontal lobe epilepsy; LCM, lacosamide; LEV, levetiracetam; LOCF, last observation carried forward; LTG, lamotrigine; OXC, oxcarbamazepine; PER, perampanel; TLE, temporal lobe epilepsy; TPM, topiramate; ZNS, zonisamide.

**TABLE 4:** Individual changes on ASMs, frequency of predominant seizure type, and EpiTrack total scores during follow‐up in patients with mildly impaired EpiTrack performance at baseline.

Sex/age at baseline	Epilepsy type	Psychiatric comorbidity	ASMs at baseline	ASMs at LOCF	Predominant seizure type	Monthly seizure frequency 12 months prior to baseline	Change in predominant seizure type from baseline	Duration of VNS therapy (months)	EpiTrack change
M/39	FLE	Yes/past	LCM 350, CBZ 1100	LCM 400↑, CBZ 1100	FIAS	7	↓ ≥ 50% < 75%	20	30 → 34
F/32	TLE	Yes/present	LTG 400, TPM 450	LTG 400, TPM 325↓	FBTCS	NA	NA	40	29 → 30
F/23	FLE	Yes/present	LEV 3000, LCM 500	LEV 3000, LCM 500	FBTCS	1	↓ 100%	25	29 → 27
M/33	TLE	Yes/present	ZNS 300, OXC 1800, PGB 225	ZNS 200↓, OXC 1800, PGB%	FAS	70	↓ ≥ 75% < 90%	35	31 → 37
F/28	FLE	No	LCM 600, BRV 200, PER 2	LCM 600, BRV 200, PER 4↑	FIAS	0.5	↓ 100%	12	31 → 32
M/50	Other	No	ESL 1600, LEV 1500, CLB 30	ESL 1600, LEV 1500, CLB 30	FIAS	4	↓ ≥ 75% < 90%	13	30 → 26
M/51	Other	No	LEV 1000, ESL 1200, PER 6	LEV 1000, ESL 1200, PER%	FAS	10	↑ > 100%	46	29 → 32
M/39	Other	No	ZNS 500, LTG 500, CLB 50	ZNS 500, LTG 400↓, CLB 40↓	FIAS	0.5	↑ ≥ 75% < 90%	40	29 → 14

*Note*: ↓ = decrease/reduction, ↑ = increase, + = add on, % = withdrawn. The blue boxes in the ‘ASMs at LOCF’ column highlights ASM decrease/reduction, while the red boxes indicate ASM increase. In the ‘Change in predominant seizure type from baseline’ column, blue highlights more than 50% seizure reduction, and red indicates more than 50% seizure increase. In the ‘EpiTrack change’ column, blue highlights patients with clinically significant improvement.

Abbreviations: ASM, antiseizure medication; BRE, brivaracetam; CBZ, Carbamazepine; CLB, clobazam; ESL, eslicarbazepine acetate; FAS, focal aware seizure; FBTCS, focal to bilateral tonic clonic seizure; FIAS, focal impaired awareness seizure; FLE, frontal lobe epilepsy; LCM, lacosamide; LEV, levetiracetam; LOCF, last observation carried forward; LTG, lamotrigine; NA, not available; OXC, oxcarbamazepine; PER, perampanel; PGB, pregabalin; TLE, temporal lobe epilepsy; TPM, topiramate; ZNS, zonisamide.

**TABLE 5:** Individual changes on ASMs, frequency of predominant seizure type, and EpiTrack total scores during follow‐up in patients with severely impaired EpiTrack performance at baseline.

Sex/age at baseline	Epilepsy type	Psychiatric comorbidity	ASMs at baseline	ASMs at LOCF	Predominant seizure type	Monthly seizure frequency 12 months prior to baseline	Change in predominant seizure type from baseline	Duration of VNS therapy (months)	EpiTrack change
F/41	FLE	No	LCM 600	LCM 600	FIAS	17	↓ ≥ 50% < 75%	31	25 → 35
F/28	FLE	No	OXC 1200, ZNS 400	OXC 1200, ZNS 250↓	FIAS	2	↓ ≥ 50% < 75%	29	18 → 24
F/38	Other	Yes/present	PER 6, CLB 20	PER 4↓, CLB 20	FBTCS	4	No change	15	14 → 25
F/19	TLE	Yes/present	OXC 2100, ZNS 400, LEV 1000	OXC 2100, ZNS 200↓, LEV%	FIAS	5	↑ ≥ 25% < 50%	29	28 → 32
F/22	TLE	No	LCM 500, BRV 150, ZNS 200	LCM 500, BRV 200↑, ZNS%	FIAS	2	↓ ≥ 75% < 90%	18	26 → 29
F/41	TLE	No	LTG 200, ZNS 300, VPA 600	LTG 200, ZNS 300, VPA 600, CLB 10+	FIAS	1	↓ 100%	60	24 → 26
M/46	FLE	No	LTG 200, VPA 1200, TPM 400	LTG 200, VPA 1200, TPM 400	FBTCS	1	↓ ≥ 50% < 75%	46	22 → 29
M/24	FLE	No	OXC 1800, PER 12, CLB 90	OXC 1800, PER 12, CLB 80↓	FAS	30	↓ < 25%	31	22 → 18
F/70	FLE	No	LTG 400, PER 10, CLB 30	LTG 400, PER 8↓, CLB%	FIAS	10	↓ < 25%	42	21 → 30
M/32	TLE	No	LTG 400, ZNS 400, CLB 40	LTG 400, ZNS 400, CLB 20↓	FIAS	4	↓ ≥ 25% < 50%	35	20 → 19
F/45	TLE	Yes/past	LCM 400, TPM 400, BRV 200	LCM 400, TPM 100↓, BRV 200	FIAS	5	↓ ≥ 25% < 50%	24	20 → 26
M/32	TLE	Yes/present	LTG 400, ZNS 500, CLB 20	LTG 400, CLB%, ZNS%	FIAS	Daily	↓ 100%	53	11 → 19
F/33	FLE	Yes/past	ZNS 500, ESL 1600, CLB 30, LEV 1500	ZNS 500, ESL 1600, LEV%, CLB%	FBTCS	1	↑ 100%	32	12 → 17

*Note*: ↓ = decrease/reduction, ↑ = increase, + = add on, % = withdrawn. The blue boxes in the ‘ASMs at LOCF’ column highlight ASM decrease/reduction, while the red boxes indicate ASM increase. In the ‘Change in predominant seizure type from baseline’ column, blue highlights more than 50% seizure reduction, and red indicates more than 50% seizure increase. In the ‘EpiTrack change’ column, blue highlights patients with clinically significant improvement.

Abbreviations: ASM, antiseizure medication; BRE, brivaracetam; CLB, clobazam; ESL, eslicarbazepine acetate; FAS, focal aware seizure; FBTCS, focal to bilateral tonic clonic seizure; FIAS, focal impaired awareness seizure; FLE, frontal lobe epilepsy; LCM, lacosamide; LEV, levetiracetam; LOCF, last observation carried forward; LTG, lamotrigine; OXC, oxcarbamazepine; PER, perampanel; TLE, temporal lobe epilepsy; TPM, topiramate; VPA, valproate; ZNS, zonisamide.

### 3.2 Effect of psychiatric comorbidities on EpiTrack performance

**FIGURE 2:**
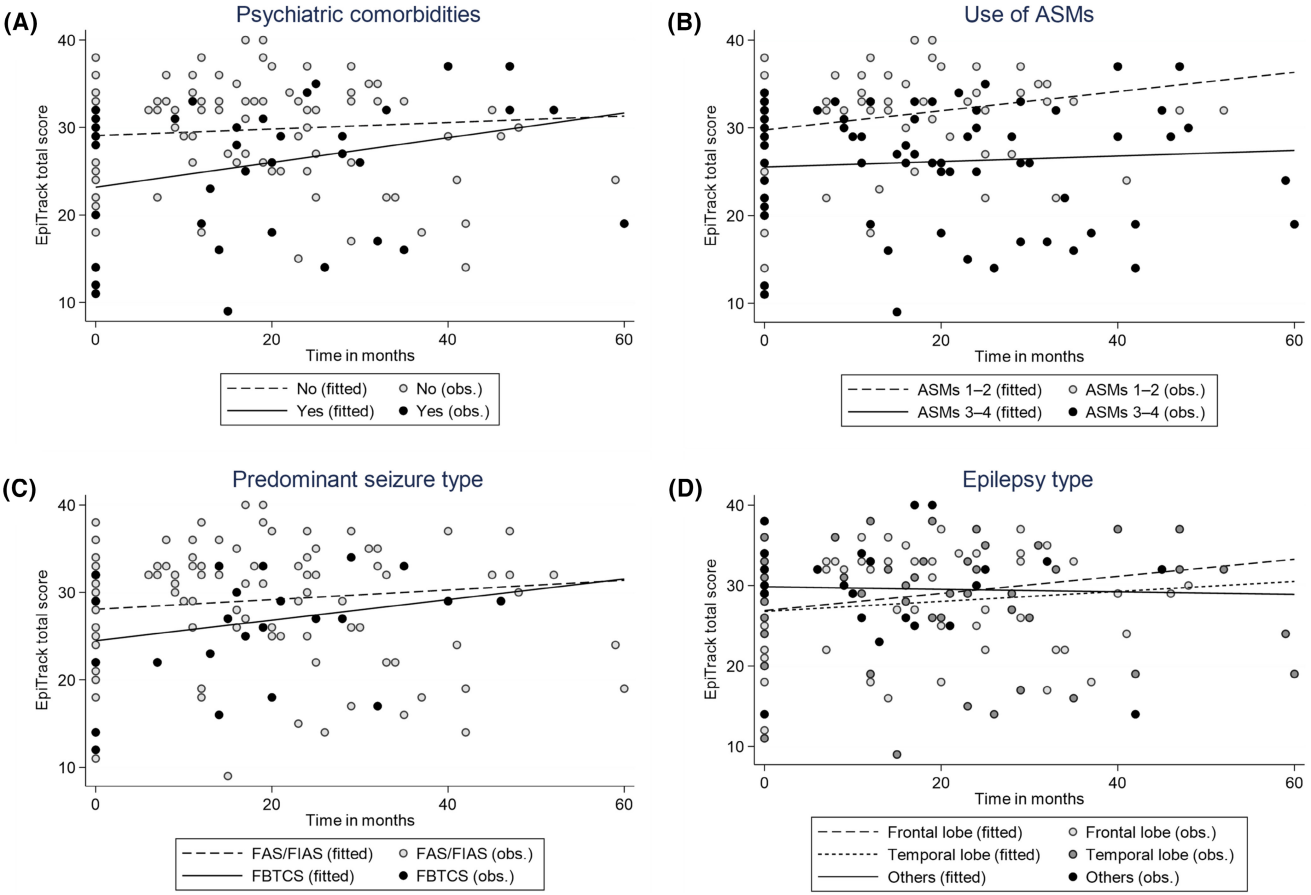
Observed EpiTrack total scores and fitted curves based on linear mixed‐effects model over time following VNS therapy based on (A) presence of psychiatric comorbidities, (B) number of ASMs in use, (C) predominant seizure types, and (D) epilepsy types. ASM, antiseizure medications; FAS, focal aware seizure; FIAS, focal impaired awareness seizure; FBTCS, focal to bilateral tonic–clonic seizure; VNS, vagus nerve stimulation. The examination was unadjusted, but any essential changes were not found in any models when age and gender were added to the model.

Before implantation, nine patients had psychiatric comorbidities (seven had depression, one had bipolar disorder, and one had psychosis), and 24 patients did not have psychiatric comorbidities. At baseline, the EpiTrack total scores of patients with psychiatric comorbidities were lower (score: 23) than those of patients without psychiatric comorbidities (score: 28.9) (95% CI: −11.8 to 0.05, *P* = 0.05) without reaching statistical significance. During the follow‐up period, the EpiTrack total score increased significantly, by an average of 0.14 units per month (95% CI: 0.049–0.23, *P* = 0.003, Figure 2A) among patients with psychiatric comorbidities, whereas patients without psychiatric comorbidities exhibited increases in the EpiTrack total score by an average of 0.04 units per month (95% CI: −0.025 to 0.10, *P* = 0.25). This corresponds to a change from a baseline score of 23 (severe impairment) to a score of 26.3 (severe impairment) at 2 years and a score of 31.4 (mild impairment) at 5 years among patients with psychiatric comorbidities compared to a change from a score of 28.9 (mild impairment) to a score of 29.8 (mild impairment) at 2 years and to a score of 31.3 (mild impairment) at 5 years among patients without psychiatric comorbidities (Table 2).

In descriptive analysis, among those 12 patients with clinically significant improvement in EpiTrack total scores, 7 (58.3%) patients had psychiatric comorbidities at baseline (Tables 3, 4, 5).

### 3.3 Effect of ASMs on EpiTrack performance

Since treatment with more than two ASMs is associated with worse cognitive performance,^16^, ^17^ we compared the change in EpiTrack total scores during follow‐up between patients taking 1–2 ASMs and 3–4 ASMs. At baseline, patients taking 1–2 ASMs (*n* = 15) had a higher EpiTrack total score (score: 29.6) than patients taking 3–4 ASMs (*n* = 18) (score: 25.3) (95% CI: −8.69 to 0.24, *P* = 0.06) without reaching statistical significance. During the follow‐up period, the EpiTrack total score increased significantly, by an average of 0.11 units per month (95% CI: 0.03–0.19, *P* = 0.005), for patients taking 1–2 ASMs, whereas an increase of only 0.03 units per month (95% CI: −0.04 to 0.11, *P* = 0.41, Figure 2B) was predicted for patients taking 3–4 ASMs. This corresponds to a change from a baseline score of 29.6 (mild impairment) to a score of 32.2 (normal) at 2 years and to a score of 36.2 (normal) at 5 years among patients taking 1–2 ASMs. In contrast, patients taking 3–4 ASMs improved from a baseline score of 25.3 (severe impairment) to a score of 26 (severe impairment) at 2 years and to a score of 27.1 (severe impairment) at 5 years (Table 2).

Treatment with topiramate (TPM) or zonisamide (ZNS), among newer generation ASMs, has also been associated with impaired cognitive performance^16^, ^17^; hence, we performed additional analysis to compare the change in EpiTrack total scores during follow‐up between patients taking TPM (*n* = 4) or ZNS (*n* = 11) and patients taking neither of these compounds in a combination of 1–2 ASMs or 3–4 ASMs. At baseline, the EpiTrack total score was the highest for the patients taking 1–2 ASMs not including TPM or ZNS (30.3) among all groups (Table S1). During the follow‐up period, the EpiTrack total score significantly increased by an average of 0.12 units per month for the aforementioned group (95% CI: 0.03–0.21, *P* = 0.009). Conversely, for patients taking 1–2 ASMs including TPM or ZNS, the EpiTrack total score increased by an average of 0.06 units per month (95% CI: −0.04 to 0.16, *P* = 0.28), for patients taking 3–4 ASMs including TPM or ZNS, the EpiTrack total score increased by an average of 0.04 units per month (95% CI: −0.04 to 0.14, *P* = 0.34), and for patients taking 3–4 ASMs not including TPM or ZNS, there was no change in the EpiTrack total score over time (95% CI: −0.11 to 0.10, *P* = 0.96) (Figure S6; Table S1).

In descriptive analysis, among those 12 patients with clinically significant improvement in EpiTrack total scores, three patients had unchanged medication, one patient had a dose increase, and eight patients had reduced ASM burden during the follow‐up period (Tables 3, 4, 5).

### 3.4 Effect of predominant seizure type on EpiTrack performance

At baseline, four (12.1%) patients had focal aware seizures (FAS), 21 (63.6%) had focal impaired awareness seizures (FIAS), and seven (21.2%) had focal to bilateral tonic–clonic seizures (FBTCS) as the predominant seizure type. Patients with FAS and FIAS were combined into a single group. One (3.1%) patient who was seizure‐free was removed from the analysis. At baseline, patients with FBTCS had a lower EpiTrack total score (score: 25.3) than patients with FAS/FIAS (score: 27.9) (95% CI: −9.59 to 2.41, *P* = 0.24) without reaching statistical significance. During the follow‐up period, the EpiTrack total score increased by an average of 0.12 units per month for patients in the FBTCS group (95% CI: −0.01 to 0.24, *P* = 0.07) and by an average of 0.06 units per month for patients in the FAS/FIAS group (95% CI: −0.01 to 0.12, *P* = 0.08, Figure 2C), neither was significant. This corresponds to a change from a baseline score of 25.3 (severe impairment) to a score of 28.1 (severe impairment) at 2 years and to a score of 32.5 (normal) at 5 years in the FBTCS group, whereas the FAS/FIAS group exhibited a change from a baseline score of 27.9 (severe impairment) to a score of 28.9 (mild impairment) at 2 years and to a score of 31.5 (almost normal) at 5 years (Table 2).

In descriptive analysis, among those 12 patients with clinically significant improvement in EpiTrack total scores, seven (58.3%) patients were responders (≥50% seizure reduction) for their predominant seizure type. In the FBTCS group three (42.9%) patients were cognitive responders and out of these three patients one (33.3%) was also a responder for the predominant seizure type. Conversely, in the FAS/FIAS group nine (36%) patients were cognitive responders, and out of these nine patients six (66.7%) were responders for their predominant seizure type (Tables 3, 4, 5).

### 3.5 Effect of epilepsy type on EpiTrack performance

Among the included patients, 14 had FLE, 12 were diagnosed with TLE, and the remaining seven were classified as having other types of epilepsy. At baseline, there was no difference in the EpiTrack total score in TLE (score 26.8) compared with FLE (score 26.9). In other types of epilepsy, the EpiTrack total score was non‐significantly higher (score 29.8; *P* = 0.33). During the follow‐up period, the EpiTrack total score increased by an average of 0.11 units per month in FLE (95% CI: 0.04–0.16, *P* < 0.001), and by an average of 0.06 units per month in TLE (95% CI: 0.01–0.11, *P* = 0.01), whereas for patients with other types of epilepsy the EpiTrack total score decreased by an average of 0.02 units per month (95% CI: −0.23 to 0.20, *P* = 0.89, Figure 2D). This corresponds to a change from a baseline score of 26.9 (severe impairment) to a score of 29.5 (mild impairment) at 2 years and to a score of 33.5 (normal) at 5 years in the FLE group, whereas patients with TLE exhibited a change from a baseline score of 26.8 (severe impairment) to a score of 28.2 (severe impairment) at 2 years and to a score of 30.4 (mild impairment) at 5 years. Patients with other types of epilepsy did not improve during the follow‐up period (Table 2).

The results of the LME models regarding the baseline EpiTrack total scores and change (pace of improvement) in all clinical characteristics at 2 and 5 years following VNS implantation are presented in Table 2.

## 4 DISCUSSION

The purpose of the present study was to investigate executive functions and attention using repeated EpiTrack evaluations in a group of DRE patients receiving VNS therapy during a follow‐up duration of up to 5 years. We identified four significant results in our study. The key finding was that based on the LME model, the EpiTrack total score improved during follow‐up at the group level. Second, in patients with psychiatric comorbidities, the EpiTrack total score demonstrated more than threefold increases compared to that in patients without psychiatric comorbidities. Third, the ASM regimen at baseline influenced the change in EpiTrack total score, since patients taking 1–2 ASMs exhibited improvements in EpiTrack scores almost quadruple that of patients taking 3–4 ASMs. Fourth, patients with FLE experienced an almost twofold increase in the EpiTrack total score compared to patients with TLE. In addition, for patients with FBTCS as the predominant seizure type, the LME model predicted a twofold higher increase in the EpiTrack total score compared to patients with FIAS/FAS; however, this difference did not reach statistical significance, probably due to the small sample size.

The overall improvement in the EpiTrack total score predicted by the LME model at 5 years after VNS implantation was 4.2 points, which at an individual level reflects clinically meaningful improvement.^15^ In addition, the performance category changed on average from severe impairment to almost normal performance. When addressing individual cognitive outcomes in the descriptive analysis we observed that among patients with normal EpiTrack performance at baseline only one out of 12 patients was a cognitive responder, i.e., experienced improvement of four points or more during the follow‐up period.^15^ Conversely, among patients with impaired EpiTrack performance at baseline, 11 (52.4%) patients had similar improvement suggesting that patients who cognitively benefited the most during VNS therapy probably had more dynamic factors lowering the baseline executive function performance. In many patients, a reduction in ASMs and/or seizure frequency was definitely contributing to the improvement in the EpiTrack total score but decrease in ASM burden and/or seizure frequency was not always concordant with improvement in the EpiTrack total score in individual patients supporting an additive direct effect of VNS on executive functions and attention.

Only a few previous clinical studies have examined the effects of VNS on cognition. Working memory and attention were investigated in a laboratory study of 20 patients with DRE treated with VNS assessed by a computer‐based test of executive functions with concurrent brain activity recording with EEG. Immediate positive effects on working memory and attention performance were demonstrated^9^ supporting direct positive effects of VNS much in line with our present study. Conversely, in a prospective randomized cross‐over study examining the effects of invasive and transcutaneous auricular VNS (taVNS) on verbal memory performance in 15 patients with DRE who conducted a word recognition memory paradigm no immediate direct effects were observed. However, improved verbal memory performance was seen after 6 weeks of VNS treatment irrespective of the acute intervention indicating a gradual short‐term effect accumulating over time.^11^ There is even more limited data on the long‐term effects of VNS on cognition. A study including over 300 pediatric epilepsy patients receiving VNS therapy showed that a relatively high proportion of VNS‐treated patients substantially improved in concentration, verbal communication, and progress in schoolwork between 12 and 24 months after initiation of VNS.^12^ Consequently, our study provides novel evidence that the executive functions of patients with DRE may improve during VNS therapy, most likely by both direct and indirect mechanisms, including changes in seizure frequency and severity, changes in the number of ASMs and improvements in mood. The possible positive effects of VNS therapy on cognition and executive functions are most likely observed over a longer time frame.^10^, ^11^, ^12^ Moreover, in our study, the duration of VNS was over 2 years in almost all cognitive responders indicating an initial timeline for expected cognitive benefits during VNS. To the best of our knowledge, no previous studies have investigated executive functions and attention in patients with DRE receiving VNS with repeated EpiTrack evaluations. Although EpiTrack was a useful screening tool in our study, it should be emphasized that EpiTrack cannot replace clinical neuropsychological testing, and further investigation with a more comprehensive approach covering all cognitive domains may be necessary.

It is well known that psychiatric comorbidities are common in patients with DRE. In our study population, almost 30% of the patients had a current or previous psychiatric comorbidity, with depression representing the most frequent (77.8%) comorbidity. Since psychiatric comorbidities are one of the most essential dynamic factors influencing cognition in DRE patients, as demonstrated in our previous study,^16^ we sought to investigate whether current or previous psychiatric comorbidities influenced the change in the EpiTrack total score during follow‐up. Among patients with psychiatric comorbidities, statistically significant improvements in the EpiTrack total score were observed, with the performance category changing from severely impaired to mildly impaired/normal at the 5‐year follow‐up. In addition, the predicted improvement in EpiTrack total score was more than threefold higher in patients with psychiatric comorbidities compared to patients without psychiatric comorbidities and twofold higher compared to that of the whole study population. Therefore, our study suggests that patients with DRE with depression experience the greatest cognitive benefit from VNS therapy, which should be taken into consideration when selecting among neuromodulation therapies.

The negative effects of ASM burden as well as specific ASMs on executive functions and cognition have been established in previous studies.^16^, ^17^, ^18^ In our preceding study,^16^ we demonstrated that among patients taking two concomitant ASMs, 40% exhibited normal performance on EpiTrack, whereas among patients taking four ASMs, only 8.3% exhibited normal performance. Our current results are in line with previous studies suggesting that treatment with more than two ASMs is associated with impaired cognitive performance.^16^, ^17^ First, patients taking 1–2 ASMs exhibited significant increases in their EpiTrack scores during follow‐up. Second, the predicted improvement in the EpiTrack total score of patients taking 1–2 ASMs during the 5‐year follow‐up period was almost quadruple that of patients taking 3–4 ASMs. Third, at baseline, the EpiTrack total score was higher among patients taking 1–2 ASMs. In addition, the selection of ASMs appeared to have a clear effect on changes in the EpiTrack total score, since patients taking 1–2 ASMs not including TPM or ZNS exhibited twofold higher EpiTrack total score improvements during follow‐up compared to patients taking 1–2 ASMs including TPM or ZNS. Both TPM and ZNS are known to impair particularly verbal fluency,^19^, ^20^ which is evaluated in one of EpiTrack subtests, but in this study the specific effect on language fluency was not evaluated separately. Our results provide additional evidence regarding treatment options in terms of cognition; that is, taking more than two ASMs, especially if including TPM/ZNS, is likely to lead to impaired cognitive performance.

Poor‐seizure control is usually associated with poor cognitive outcomes related to both seizure frequency and severity.^21^ In our descriptive analysis, we observed that at baseline, patients with FBTCS as the predominant seizure type had lower EpiTrack total scores than patients with FAS/FIAS as the predominant seizure type. Conversely, patients in the FBTCS group had a twofold higher increase in EpiTrack total scores during follow‐up compared to patients in the FAS/FIAS group, without reaching statistical significance. Previously, it has been proposed that FBTCS is more likely to influence cognition in a negative manner than other seizure types.^21^, ^22^ However, in the present study, there were actually fewer responders (≥50% reduction in seizure frequency) in FBTCS group than in the FIAS/FAS group among patients with significant EpiTrack improvement (cognitive responders) suggesting that the positive correlation of EpiTrack improvement in patients with FBTCS was not solely dependent on decreased seizure frequency. Furthermore, the issue with the interplay between seizure burden and executive functions is complicated by additional factors: (i) absolute seizure count for FIAS has been demonstrated to influence EpiTrack performance,^15^ (ii) frequency of FBTCS has been shown to have a trend for EpiTrack performance,^15^ (iii) there may be changes in seizure severity and postictal recovery that can also diminish the negative effects of both FBTCS and FIAS on executive functions.

The main static factors influencing the cognitive functioning of epilepsy patients are epilepsy type and etiology. While the etiology of epilepsy was very heterogeneous in our study population, most of our study patients were diagnosed with either frontal or temporal lobe epilepsy. At baseline, EpiTrack performance did not differ between TLE and FLE. However, during follow‐up patients with FLE had almost twofold higher increase in EpiTrack total scores compared to patients with TLE. In a clinical study evaluating neuropsychological domains in patients with FLE, language was the most common domain‐specific impairment, followed by attention, executive function, and processing speed.^23^ There is data suggesting that VNS has direct effects on attention‐related event potentials,^9^ which mechanism may be more relevant for patients with FLE compared to TLE.

There is increasing data to support the direct neurobiological effects of VNS on cognition‐related brain functions.^12^, ^24^ VNS affects excitability in cognition‐associated pathways by altering brain neurotransmitters, such as gamma‐aminobutyric acid and glutamate as well as the neuromodulators serotonin, dopamine, and norepinephrine.^25^, ^26^ Activation of the nucleus of the solitary tract in the brainstem by vagal afferent nerve fibers allows for modulation of various higher‐order brain regions, e.g. the prefrontal cortex as well as processes improving alertness, arousal, short‐term memory, verbal memory recognition, working memory, mood, and decision‐making in patients with DRE or depression.^9^, ^11^, ^26^ All these activities are instrumental for executive functions. Moreover, recent preclinical studies have identified hippocampal synaptic plasticity pathways that may contribute to VNS‐induced memory enhancement,^27^ which was not evaluated in the present study.

The main limitations of our study were that it was not entirely conducted according to the prospectively defined protocol and it was not randomized. First, due to the COVID‐19 pandemic, the scheduled visits did not always take place according to our clinical VNS follow‐up protocol, with a mean follow‐up duration of 29 months. Therefore, changes in EpiTrack total scores over time were analyzed using a statistical model to compensate for the variation in time points and the numbers of EpiTrack evaluations of individual patients when predicting EpiTrack total score changes per month during a period up to 60 months. Accordingly, the changes in EpiTrack total scores presented in this study are predictions based on the LME model and not actual cohort measurements at a given time‐point after VNS implantation. In addition, the LME model did not take into account possible modifications to ASMs or seizure status changes during the follow‐up period. On the other hand, the use of the LME model yielded a statistically robust evaluation of EpiTrack total scores as time series data after VNS implantation. In addition, the practice effect may have potentially influenced scores on the retests; however, in the development of EpiTrack testing, not only the measurement error in the test–retest setting but also the practice effects were taken into consideration.^15^ Originally, the EpiTrack test was developed for the assessment of intraindividual, not group‐level changes. Moreover, for EpiTrack usage, there is no data on more than two time points or re‐assessments after several years as was the case in some of the patients included in the present study. Furthermore, the EpiTrack total score is composed of scores on the different subtests, and in the future, it might be more informative to assess performance on individual subtests. Finally, patients were not simultaneously evaluated regarding mood and executive functions.

## 5 CONCLUSION

A gradual improvement representing a clinically meaningful change in the executive functions of patients with DRE after the initiation of VNS therapy was observed. Patients with DRE with depression seemed to experience the greatest cognitive benefits from VNS therapy, which should be taken into consideration when selecting among neuromodulation therapies. The number of ASMs also had a clear effect on the cognitive response to VNS therapy, with the best outcome achieved in patients taking fewer than three ASMs.

## AUTHOR CONTRIBUTIONS

Niina Lähde: study concept and design, acquisition of data, analysis and interpretation, drafting the manuscript. Pabitra Basnyat: acquisition of data, analysis, and interpretation, and revised manuscript. Jani Raitanen: data analysis and interpretation, revised manuscript, and statistical analyses. Kai Lehtimäki: Study concept and design, revised manuscript, study supervision. Eija Rosti‐Otajärvi: Acquisition of data, analysis, and interpretation, revised manuscript. Jukka Peltola: study concept and design, critical revision, study supervision. All the authors read and approved the final manuscript.

## CONFLICT OF INTEREST STATEMENT

Niina Lähde has participated in a clinical trial for UCB; and received speaker honoraria from LivaNova (OmaMedical). Jani Raitanen has participated in clinical trials for Eisai, UCB, and Bial; received research grants from Angelini Pharma, Eisai, Medtronic, UCB, and LivaNova; received speaker honoraria from LivaNova, Angelini Pharma, Eisai, Jazz Pharma, Medtronic, Orion Pharma, and UCB; received support for travel to congresses from LivaNova, Eisai, Medtronic, and UCB; and participated in advisory boards for LivaNova, Angelini Pharma, Jazz Pharma, Eisai, Medtronic, UCB, and Pfizer. Kai Lehtimäki has received speaker honoraria from Medtronic. Eija Rosti‐Otajärvi has received speaker honoraria from Novartis and Biogen. The remaining authors have no conflicts of interest.

## ETHICS STATEMENT

This was a non‐interventional study in which data was collected prospectively but analyzed retrospectively from a VNS quality register at Tampere University Hospital, therefore not requiring ethics committee approval according to Finnish Law on Research.

## PATIENT CONSENT STATEMENT

Not needed.

## Supporting information


Figure S1.

Table S1.

## Data Availability

The data that support the findings of this study are available on request from the corresponding author. The data are not publicly available due to privacy or ethical restrictions.
