# Supplementary material for: Longitudinal EpiTrack assessment of executive functions following vagus nerve stimulation therapy in patients with drug‐resistant epilepsy
Source: Epilepsia Open. 2023 Nov 27;9(1):150–63. doi: 10.1002/epi4.12855 (PMC10839331; doi:10.1002/epi4.12855)

Epilepsia Open

Supporting Information

Longitudinal EpiTrack assessment of executive functions following vagus nerve stimulation therapy in patients with drug-resistant epilepsy

Niina Lähde[1,2], Pabitra Basnyat[2], Jani Raitanen[3], Kai Lehtimäki[4], Eija Rosti-Otajärvi[1,5], Jukka Peltola[1,2]

[1] Department of Neurology, Tampere University Hospital, Tampere, Finland

[2] Faculty of Medicine and Health Technology, Tampere University, Tampere, Finland

[3] Faculty of Social Sciences, Health Sciences, Tampere University, Tampere, Finland; UKK Institute for Health Promotion Research, Tampere, Finland

[4] Department of Neurosurgery, Tampere University Hospital, Tampere, Finland

[5] Department of Rehabilitation and psychosocial support, Tampere University Hospital, Tampere, Finland

Corresponding author

Niina Lähde, MD

Tampere University Hospital

Department of Neurology

Elämänaukio 2,

33520 Tampere, Finland

Email: niina.lahde@pirha.fi; nlahde@gmail.com

Tel: +358 452 392 308

Supplementary fig 1. The flowchart of the follow-up protocol

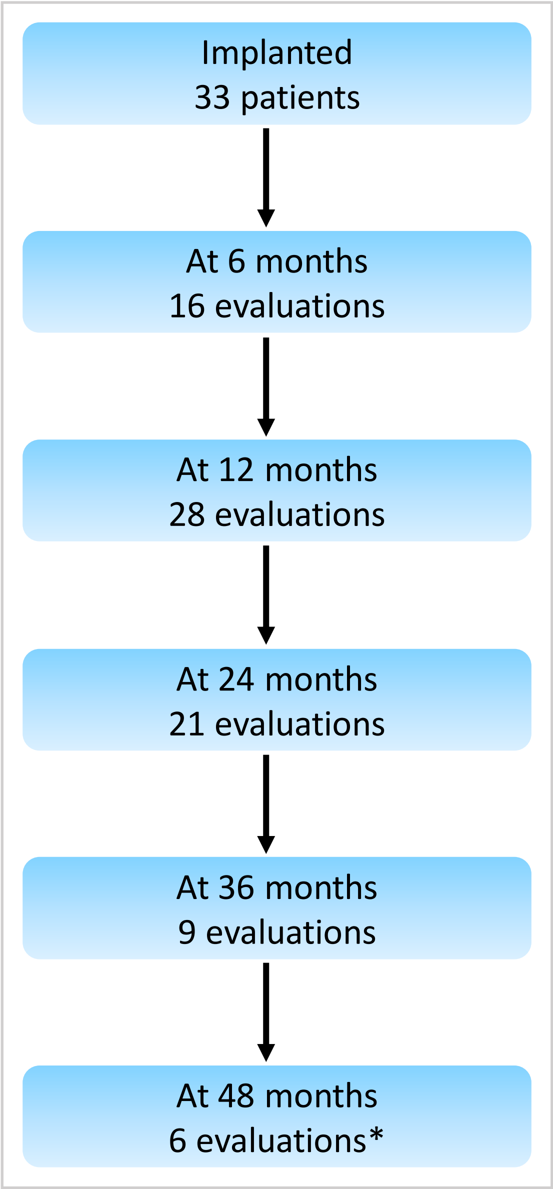

The figure includes summary of EpiTrack evaluations at different time points after VNS implantation. Each time point represents a specific duration in months as follows: 6 months (ranging from 4 to 8 months), 12 months (ranging from 18 to 30 months), 24 months (ranging from 30 to 42 months), and 36 months (ranging from 42 to 54 months). * Two evaluations at 61 and 70 months are included in this time point.

Supplementary fig 2. Patients using 1-2 ASM at baseline and their ASM status and EpiTrack changes during the follow-up.

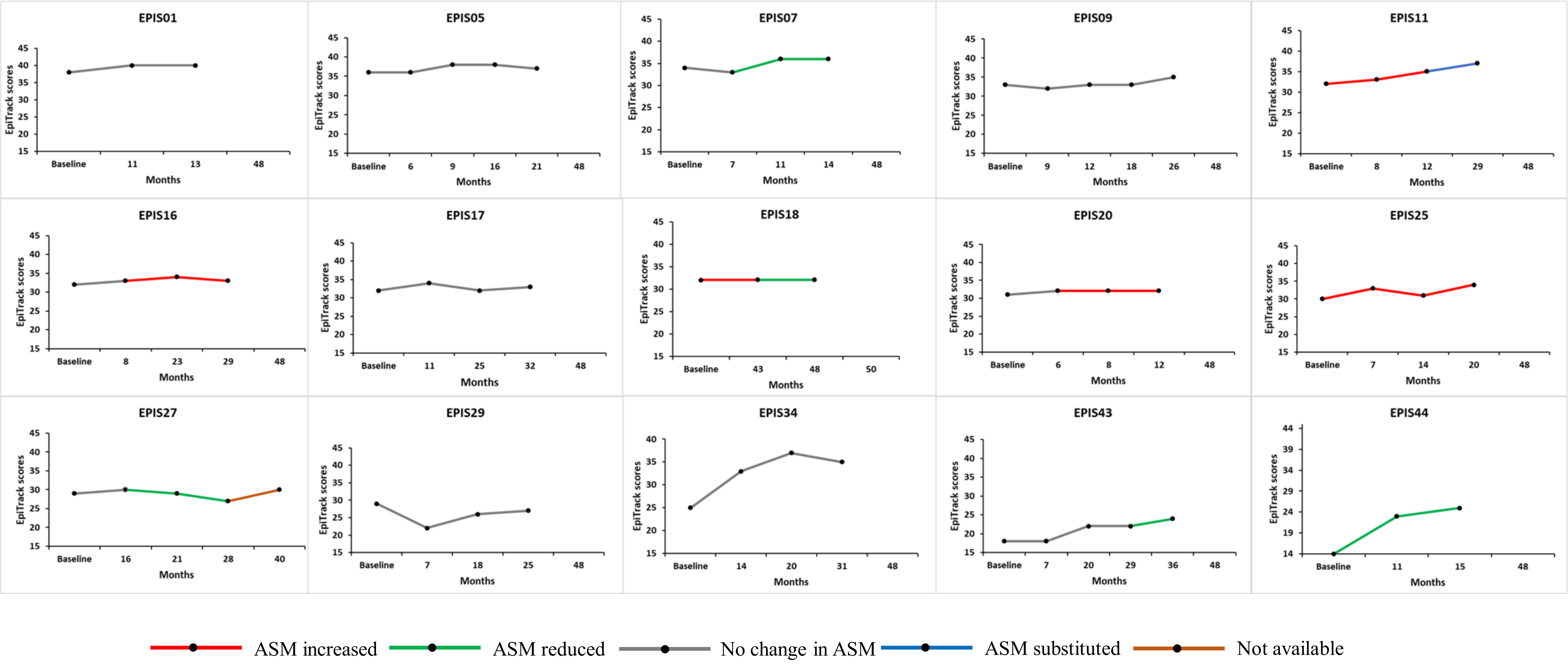

Supplementary fig 3. Patients using 3-4 ASM at baseline and their ASM status and EpiTrack changes during the follow-up.

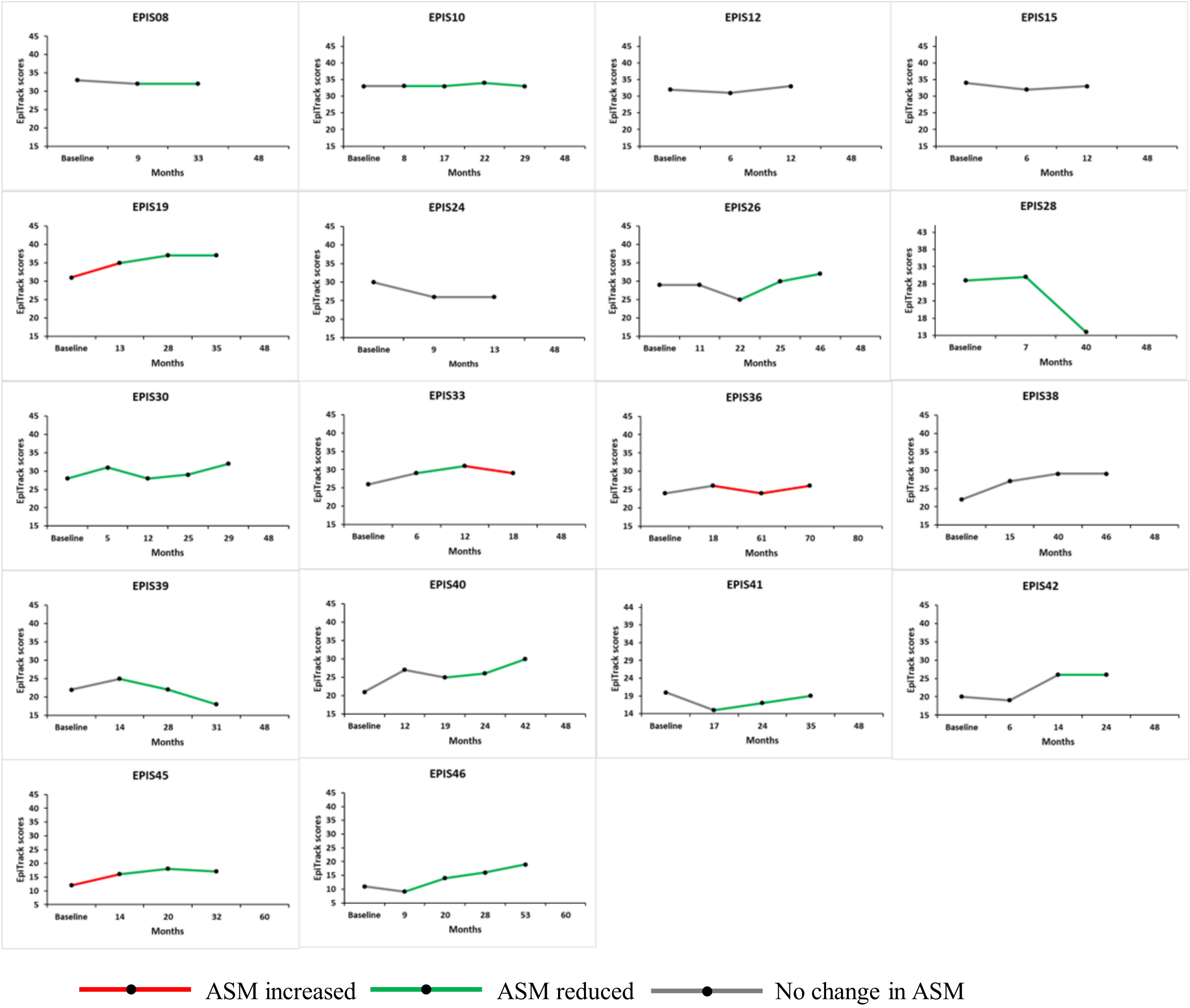

Supplementary fig 4. Patients with FAS/FIAS as the predominant seizure type at baseline and their seizure status and EpiTrack changes during the follow-up.

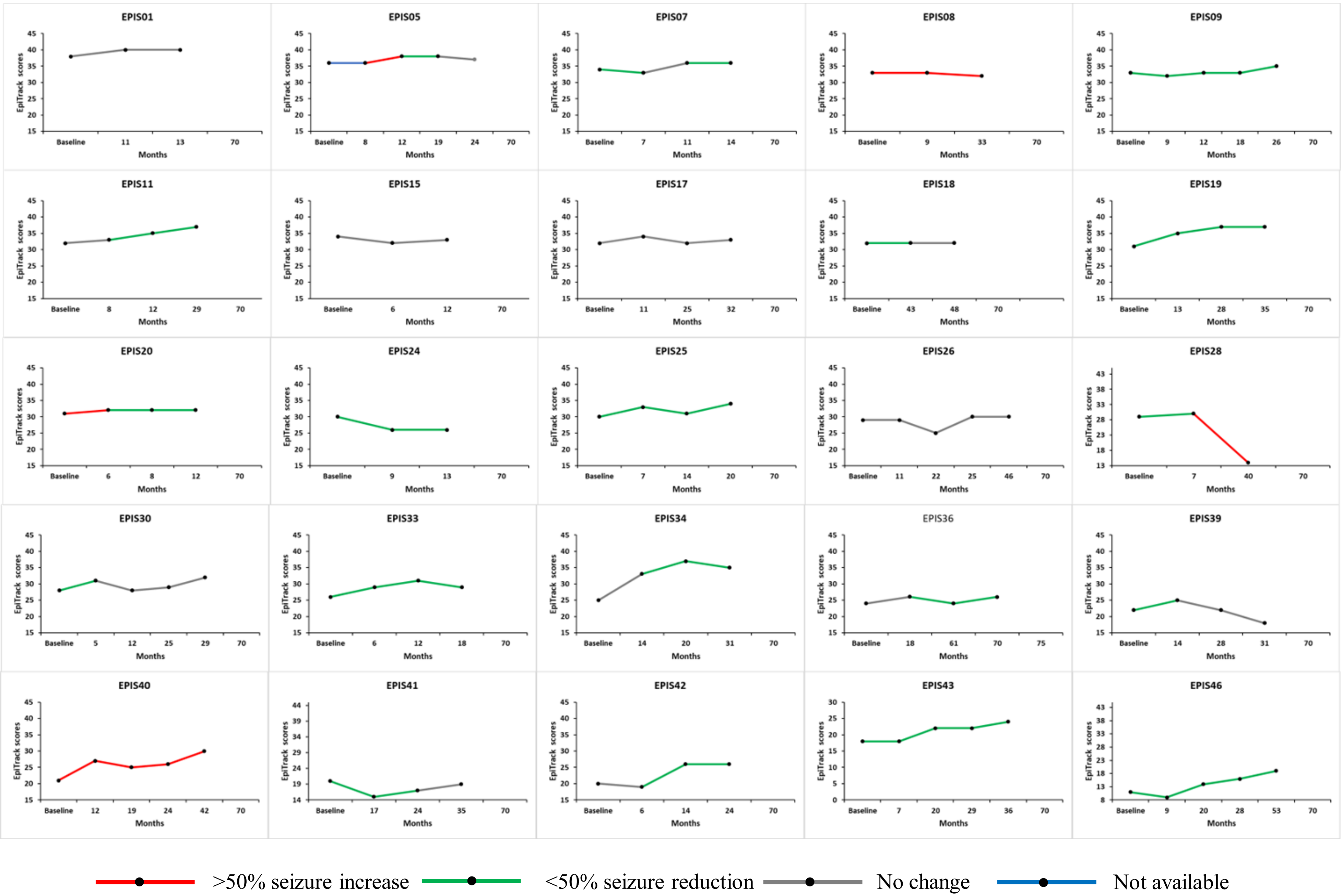

Supplementary fig 5. Patients with FBTCS as the predominant seizure type at baseline and their seizure status and EpiTrack changes during the follow-up.

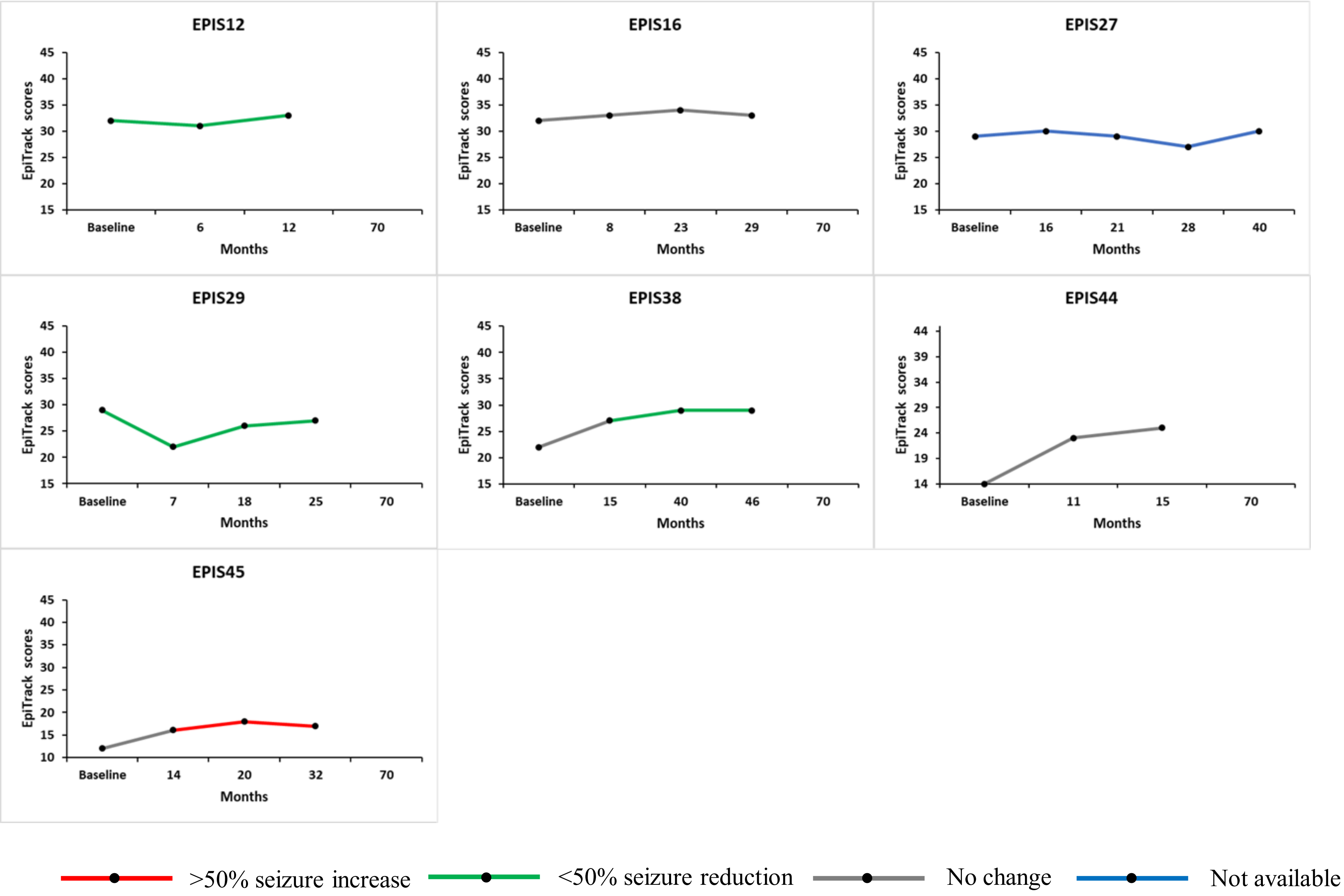

Supplementary fig 6. Observed EpiTrack total scores and fitted curves based on linear mixed-effects model over time following VNS therapy based on different types of ASMs; ASMs with or without TPM (topiramate) or ZNS (zonisamide).

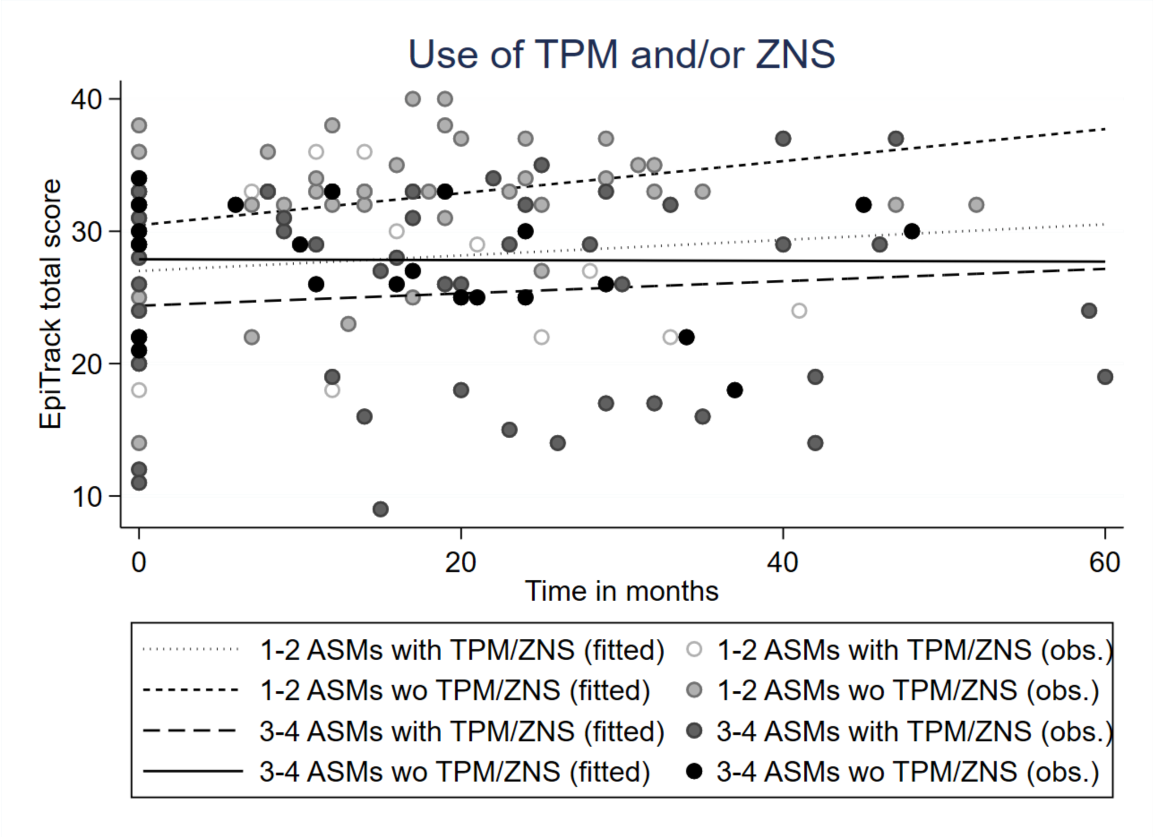

Supplement: Supplementary file 1 — Figure S1. [file EPI4-9-150-s001.pdf]
