# Supplementary material for: Longitudinal EpiTrack assessment of executive functions following vagus nerve stimulation therapy in patients with drug‐resistant epilepsy
Source: Epilepsia Open. 2023 Nov 27;9(1):150–63. doi: 10.1002/epi4.12855 (PMC10839331; doi:10.1002/epi4.12855)
Supplement: Supplementary file 2 — Table S1. [file EPI4-9-150-s002.docx]

**Supplementary Table 1.** Baseline EpiTrack total scores and change (pace of improvement) for ASM groups based on the use of TPM or ZNS at 2-years and 5-years after the VNS implantation

| ASMs | Baseline EpiTrack score | Average increase at 2 years* | Average increase at 5 years* |
| --- | --- | --- | --- |
| 1-2 ASMs with TPM/ZNS | 27.0 | 1.4 | 3.6 |
| 1-2 ASMs without TPM/ZNS | 30.3 | 2.8 | 7.2 |
| 3-4 ASMs with TPM/ZNS | 24.0 | 0.9 | 2.4 |
| 3-4 ASMs without TPM/ZNS | 28.0 | 0 | 0 |

*Average increase in EpiTrack scores at 2-years and 5-years were calculated as average change in EpiTrack score per month obtained from LME model divided by 24 months and 60 months. ASM, antiseizure medication; TPM, topiramate; ZNS, zonisamide.
